# Validation of the STOP-Bang questionnaire as a preoperative screening tool for obstructive sleep apnea: a systematic review and meta-analysis

**DOI:** 10.1186/s12871-022-01912-1

**Published:** 2022-11-30

**Authors:** Mark Hwang, Mahesh Nagappa, Nasimi Guluzade, Aparna Saripella, Marina Englesakis, Frances Chung

**Affiliations:** 1 Faculty of Medicine, University of British Columbia, Vancouver, BC V6T 1Z3 Canada; 2 Department of Anesthesia and Pain Medicine, Toronto Western Hospital, University Health Network, University of Toronto, Toronto, ON M5T 2S8 Canada; 3 Department of Anesthesia & Perioperative Medicine, London Health Sciences Centre and St. Joseph Health Care, Schulich School of Medicine and Dentistry, Western University, London, ON Canada; 4 Library and Information Services, University Health Network, Toronto, ON Canada; 5 Department of Anesthesiology and Pain Management, University Health Network, University of Toronto, MCL 2-405, 399 Bathurst Street, Toronto, ON M5T 2S8 Canada

**Keywords:** Obstructive sleep apnea, Screening questionnaire, STOP-bang questionnaire, Surgical population, Systematic review, Meta-analysis

## Abstract

**Background:**

Obstructive sleep apnea (OSA) is a common disorder that is highly associated with postoperative complications. The STOP-Bang questionnaire is a simple screening tool for OSA. The objective of this systematic review and meta-analysis is to evaluate the validity of the STOP-Bang questionnaire for screening OSA in the surgical population cohort.

**Methods:**

A systematic search of the following databases was performed from 2008 to May 2021: MEDLINE, Medline-in-process, Embase, Cochrane Central Register of Controlled Trials, Cochrane Database of Systematic Reviews, PsycINFO, Journals @ Ovid, Web of Science, Scopus, and CINAHL. Continued literature surveillance was performed through October 2021.

**Results:**

The systematic search identified 4641 articles, from which 10 studies with 3247 surgical participants were included in the final analysis. The mean age was 57.3 ± 15.2 years, and the mean BMI was 32.5 ± 10.1 kg/m^2^ with 47.4% male. The prevalence of all, moderate-to-severe, and severe OSA were 65.2, 37.7, and 17.0%, respectively. The pooled sensitivity of the STOP-Bang questionnaire for all, moderate-to-severe, and severe OSA was 85, 88, and 90%, and the pooled specificities were 47, 29, and 27%, respectively. The area under the curve for all, moderate-to-severe, and severe OSA was 0.84, 0.67, and 0.63.

**Conclusions:**

In the preoperative setting, the STOP-Bang questionnaire is a valid screening tool to detect OSA in patients undergoing surgery, with a high sensitivity and a high discriminative power to reasonably exclude severe OSA with a negative predictive value of 93.2%.

**Trial registration:**

PROSPERO registration CRD42021260451.

**Supplementary Information:**

The online version contains supplementary material available at 10.1186/s12871-022-01912-1.

## Background

Obstructive sleep apnea (OSA) is the most common sleep-related breathing disorder, characterized by episodes of apnea and hypopnea [1]. Recognizing OSA in undiagnosed patients preoperatively is particularly important, as many analgesics, anesthetics, and sedatives are respiratory depressants that can exacerbate OSA [1, 2]. Up to 68% of patients undergoing surgery with OSA can be undiagnosed, [2, 3] resulting in increased risk of perioperative cardiovascular and pulmonary complications [2, 4–7]. Thus, an easy-to-administer screening tool for preoperative assessment of patients undergoing surgery at increased risk for OSA is essential in the armament of perioperative risk stratification.

Polysomnography (PSG) is the diagnostic standard for OSA. However, PSG can be difficult to access as it is expensive, time consuming, and requires overnight laboratory observation [8]. With limited resources for PSG and the high prevalence of OSA in the general population, several screening tools have been developed for clinicians to prioritize diagnosis and treatment in patients with increased risk of OSA. In the surgical population, the STOP-Bang questionnaire, [9] STOP questionnaire, [9] P-SAP score, [10] Berlin questionnaire, [11] and ASA checklist [11] are validated screening tools for OSA.

The STOP-Bang questionnaire is a simple tool for detecting OSA that takes approximately 1 minute to complete. It has been validated in multiple settings [9, 12–15] and used worldwide in different populations [13–16]. The STOP-Bang questionnaire consists of four binary (STOP: **S**noring, **T**iredness, **O**bserved apnea, and high blood **P**ressure), and four demographic questions (Bang: **B**ody mass index (BMI), **a**ge, **n**eck circumference, and **g**ender) [9]. When first developed, the STOP-Bang questionnaire with a cut-off score of 3 or greater had demonstrated a sensitivity of 83.9, 92.9, and 100% in detecting all OSA (Apnea–Hypopnea Index (AHI) ≥5 events per hour), moderate-to-severe OSA (AHI ≥15 events per hour), and severe OSA (AHI ≥30 events per hour), respectively [9]. As a preoperative diagnosis of OSA is associated with higher risk of complications in the perioperative setting, [4, 17–19] the predictive parameters of the STOP-Bang questionnaire in the surgical population should be evaluated to determine its utility in predicting perioperative complications associated with OSA. To date, no systematic review and meta-analysis has examined the validity of the STOP-Bang questionnaire to detect OSA specifically in the surgical population. The objective of this systematic review and meta-analysis is to determine the validity of the STOP-Bang questionnaire as a preoperative screening tool in identifying those at increased risk of OSA in the surgical population cohort.

## Methods

### Study registration

The protocol of this study was registered in the International Prospective Register of Systematic Reviews (PROSPERO; registration CRD42021260451). The study was completed in accordance with the Preferred Reporting Items for Systematic Reviews and Meta-analyses (PRISMA) guideline [20].

### Literature search strategy

The literature search was performed by an information specialist (ME) using the Ovid platform for the following databases: MedlineALL, Embase, Cochrane Central Register of Controlled Trials, Cochrane Database of Systematic Reviews, APA PsycINFO, and Journals@Ovid. CINAHL, the Web of Science, and Scopus (Elsevier) were also searched. Search components consisted of (“stop-bang” or “stopbang”) AND (perioperative or postoperative or surgery) related terms. Searches were limited to the years 2008 (development of STOP-Bang questionnaire) to May 14, 2021. No other limits were applied. Literature surveillance was performed through November 2021. The Medline search strategy is provided in the supplemental material (Appendix 1).

### Study selection and data management

Title and abstract screening, and full text evaluation were independently completed by two reviewers (MH, NG) using Covidence. Full text articles meeting the following inclusion criteria were included: 1) the study screened for OSA using the STOP-Bang questionnaire in adult patients aged ≥18 years undergoing surgery; 2) OSA diagnosis confirmed by PSG or home sleep apnea testing (HSAT); 3) severity of OSA measured by Apnea Hypopnea Index (AHI) or Respiratory Disturbance Index (RDI) cut-offs ≥5, ≥ 15, and ≥ 30 events per hour; and 4) accuracy of the STOP-Bang questionnaire assessed with predictive parameters. The two reviewers extracted data from the included studies with a standardized form. A third reviewer (AS) resolved any discrepancy between the reviewers. Data collection was managed in Excel (Redmond, United States).

### Evaluation of methodological quality

Two reviewers (MH, NG) independently evaluated bias of the included studies. The assessment was conducted using criteria for internal and external validity coded using the Cochrane Screening and Diagnostic Tests Methods Group [21]. The result of the evaluation was compared and a third reviewer (AS) resolved any discrepancies. Internal validity was assessed using the following criteria: valid reference standard, definition of disease, blind execution of index and reference tests, interpretation of index test independent of clinical information, and study design. External validity was assessed using the following criteria: disease spectrum, clinical setting, previous screening or referral filter, demographic information, explicit cut-off of index test, percentage of missing participants, management of missing data, and selection of participants for the reference test. Furthermore, the Quality Assessment of Diagnostic Accuracy Studies (QUADAS) tool was used by the reviewers to rate the quality of individual included study on a scale ranging from 0 to 14 [22].

### Statistical analysis

Meta-analysis was performed with Review Manager Version 5.4 and Meta-disc V.1.4. For each of the included studies, 2 × 2 contingency tables were created to obtain predictive parameters with 95% confidence intervals. The following pooled predictive parameters were calculated using a bivariate random-effects model: sensitivity, specificity, positive predictive value (PPV), negative predictive value (NPV), diagnostic odds ratio (DOR), likelihood ratios, and area under the curve (AUC) to evaluate the validity of the STOP-Bang questionnaire for different OSA severities defined by AHI cut-offs: AHI ≥ 5 (all), AHI ≥ 15 (moderate-to-severe), and AHI ≥ 30 (severe) events per hour. A STOP-Bang score of three or greater was accepted as the threshold and post-test probabilities were calculated as described by Brooks et al. [23] Also, the pooled predictive parameters of additional STOP-Bang score thresholds were calculated for different OSA severities.

Meta-regression and sensitivity analysis were performed for moderate-to-severe and severe OSA using Open Meta Analyst software [24] for categorical variables (validation tools and study type) and continuous variables (age, male gender, BMI, neck circumference, prevalence, and sample size). We focused to measure the association between these variables and the combined estimates of sensitivity, specificity, and log scale diagnostic odds ratio. Leave-one-study-out analysis was performed to examine the effect, if any, of individual study on the reliability of the combined estimates. Level of statistical significance was set at *p* value < 0.05.

## Results

**Fig. 1:**
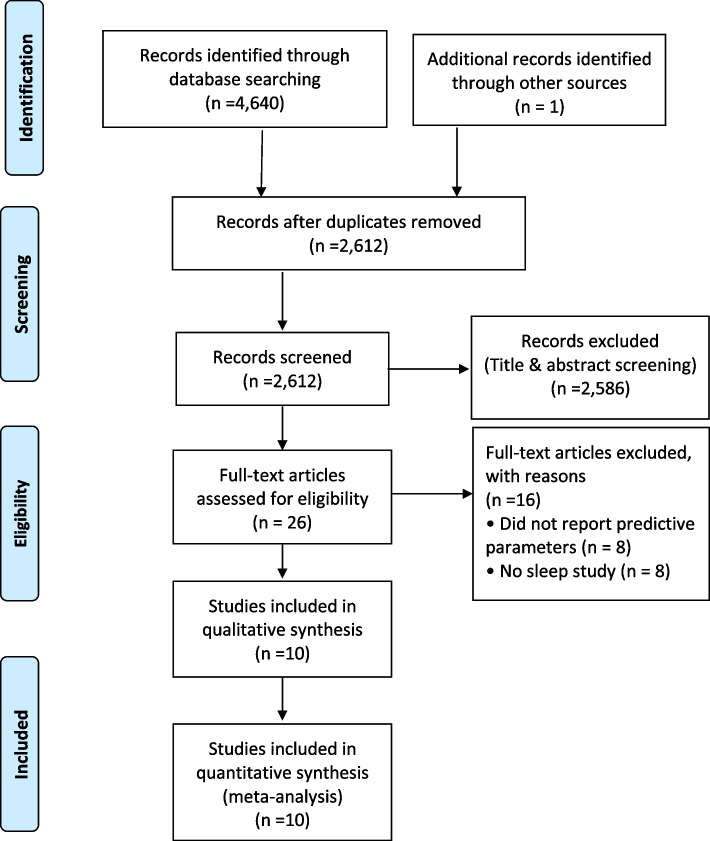
PRISMA Flow Diagram

The search of literature identified 4641 articles, from which 2029 duplicates were removed (Fig. 1). Following the review of titles and abstracts, 2586 studies did not meet the inclusion criteria and were excluded. The full text of the remaining 26 studies were reviewed, and 16 full text articles [25–40] were excluded due to reasons listed in Supplementary Table S1. The review included 10 articles that satisfied the inclusion criteria [41–50]. Of note, Nunes et al. [42] and Waseem et al. [50] included two and four subgroups, respectively, yielding a total of 14 included study groups. The included studies involved 3247 surgical patients who were preoperatively evaluated for OSA.

**Fig. 2:**
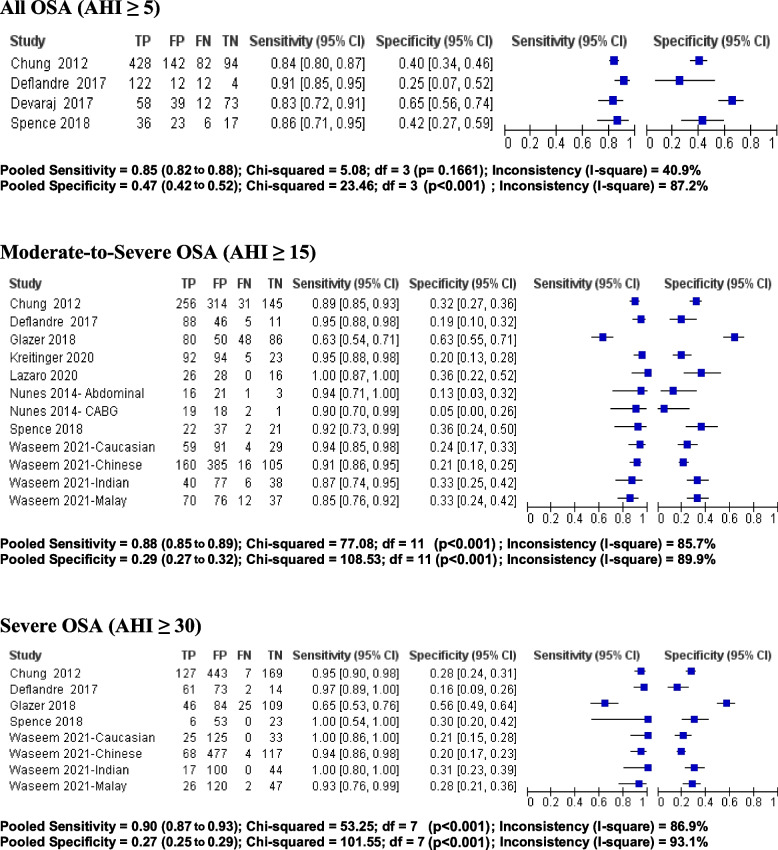
Forest plot of pooled sensitivity and specificity of STOP-Bang questionnaire for various OSA severities in surgical patients. Values are presented as means with 95% CI in parentheses. Abbreviations: AHI, Apnea–Hypopnea index; Bang, body mass index, age, neck circumference and gender; CABG, coronary artery bypass graft surgery; CI, confidence interval; OSA, obstructive sleep apnea; STOP, snoring, tiredness, observed apnea and high blood pressure

**Fig. 3:**
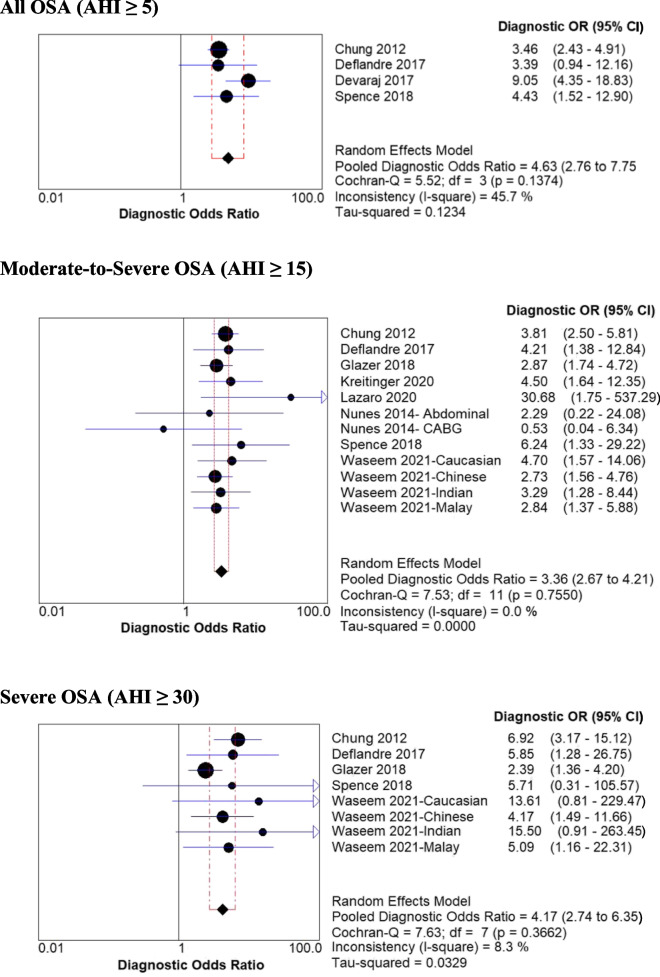
Forest plot of pooled diagnostic odds ratio of STOP-Bang questionnaire for various OSA severities in surgical patients. Values are presented as means with 95% CI in parentheses. Abbreviations: AHI, Apnea–Hypopnea index; Bang, body mass index, age, neck circumference and gender; CABG, coronary artery bypass graft surgery; CI, confidence interval; OR, odds ratio; OSA, obstructive sleep apnea; STOP, snoring, tiredness, observed apnea and high blood pressure

The demographic data of the included studies are summarized in Table 1. In surgical patients, the mean age was 57.3 ± 15.2 years, mean BMI was 32.5 ± 10.1 kg/m^2^, and 47% were male. Seven studies [41–44, 47, 49, 50] were of prospective design and three studies were retrospective [45, 46, 48]. The characteristics of the included studies are summarized in Table 2. Surgical procedures comprised of non-cardiac elective surgery [41, 44, 50] (*n* = 3), abdominal surgery [42] (*n* = 1), coronary artery bypass grafting [42] (n = 1), bariatric surgery [45, 46, 48, 49] (*n* = 4), and total joint arthroplasty [47] (n = 1). The study by Nunes et al. had two different populations of surgical patients (abdominal surgery and coronary artery bypass grafting) [42]. Among the surgical population, four studies included AHI cut off ≥5 [41, 43, 44, 47], eight included AHI cut off ≥15 [41–43, 45, 47–50], and five included cut off AHI ≥30 events per hour [41, 43, 45, 47, 50] (Figs. 2 and 3).

**Table 1:** Demographic data of patients using STOP-Bang questionnaire

Study ID (Country)	Study Type	No. of patients (Surgery Type)	Age (Year)	Gender: Male/Female	BMI (Kg/m^2^)	Neck Circumference (cm)	STOP-Bang Score	AHI (mean)	Minimum SPO2 (%)
Chung 2012 [41] (Canada)	PC	746 (non-cardiac surgery)	59.7 ± 12.6	365/381	30.3 ± 6.7	39.0 ± 4.5	NR	NR	NR
Nunes 2014- Abdominal [42] (Brazil)	PC	41 (abdominal surgery)	56.0 ± 8.0	28/13	29 ± 5	39.0 ± 4	NR	19 ± 18	85.3 + 4.6
Nunes 2014- CABG [42] (Brazil)	PC	40 (CABG)	56.0 ± 7.0	29/11	30 ± 4	40 ± 4	NR	21 ± 19	85.3+ 8.5
Deflandre 2017 [43] (Belgium)	PC	150 (Pre-operative patients; surgery type not specified)	59.7 + 12.4	105/45	32.4 + 2.3	42.0 + 4.6	NR	NR	NR
Devaraj 2017 [44] (India)	PC	OSA: 70 (non-cardiac surgery)	52.7 + 11.5	36/34	27.2 + 5.0	37.1+ 4.2	NR	NR	NR
		Non-OSA: 112 (non-cardiac surgery)	46.55 + 15.5	47/65	24.1 + 4.0	34.8 + 3.7	NR	NR	NR
Glazer 2018 [45] (Canada)	RC	264 (bariatric surgery)	44.2 + 11.4	39/225	49.2 + 8.8	42.6 + 4.3	3.5 + 1.4	23.0 + 25.8	NR
Horvath 2018 [46] (Switzerland)	RC	251 (bariatric surgery)	39.0 + 13.4	60/191	42.2 + 5.7	41.7 + 4.5	3 (2 to 4)	NR	NR
Spence 2018 [47] (United States)	PC	84 (THA/TKA)	57 ± 11.8	49/35	30.3 ± 4.7	39.9 ± 4.2	3.7 ± 1.7	10.9 ± 11.9	NR
Kreitinger 2020 [48] (United States)	RC	214 (bariatric surgery)	40.6 ± 11.2	36/178	47.4 ± 8.6	48 ± 9.7	NR	21.2 ± 26.2	80.4 ± 10.2
Lazaro 2020 [49] (Spain)	PC	70 (bariatric surgery)	44.3 ± 8.8	24/46	42.4 ± 4.4	42.1 ± 3.9	4.6 ± 1.3	18.81 ± 22.4	NR
Waseem 2021- Caucasian [50] (Canada)	PC	183 (non-cardiac surgery)	70.7 ± 10.3	103/80	30.1 ± 5.5	39.3 ± 3.4	4 ± 1.4	Median: 9 IQR [4–19]	77.6 ± 10.9
Waseem 2021- Chinese [50] (Canada)	PC	666 (non-cardiac surgery)	67.5 ± 8.8	441/225	24.9 ± 4.1	38.4 ± 3.2	3.7 ± 1.3	Median: 7 IQR [3–15]	78 ± 10.3
Waseem 2021- Indian [50] (Canada)	PC	161 (non-cardiac surgery)	65.9 ± 9.7	76/85	27.4 ± 5.1	37.8 ± 3.7	3.4 ± 1.4	Median: 8 IQR [4–16]	73.5 ± 13
Waseem 2021- Malay [50] (Canada)	PC	195 (non-cardiac surgery)	64.0 ± 8.3	101/94	27.9 ± 6.2	38.8 ± 4.1	3.4 ± 1.3	Median: 11 IQR [3–22]	76 ± 11.2

Data represented as mean ± standard deviation where appropriate. *Abbreviations: AHI* apnea–hypopnea Index, *Bang* body mass index, age, neck circumference, gender, *BMI* body mass index, *CABG* coronary artery bypass graft surgery, *IQR* interquartile range, *NR* Not reported, *OSA* obstructive sleep apnea, *PC* prospective cohort, *RC* retrospective cohort, *STOP* snoring, tiredness, observed apnea and high blood pressure, *THA* total hip arthroplasty, *TKA* total knee arthroplasty

**Table 2:** Characteristics of included studies

Study ID (Country)	Study population	Validation process & Tool	OSA definition	Prevalence n (%)	No OSA AHI < 5 n (%)	Mild OSA AHI ≥5 to < 15 n (%)	Moderate OSA AHI ≥15 to < 30 or RDI > 15 to < 30 n (%)	Severe OSA AHI ≥30 or RDI ≥30 n (%)	QUADAS Score ^a^
Chung 2012 [41] (Canada)	746 (non-cardiac surgery)	Lab PSG and HSAT (Embletta X100)	AHI > 5	510 (68.4)	236 (31.6)	223 (29.9)	153 (20.5)	134 (18.0)	13
Nunes 2014 [42] (Brazil)	41 (abdominal surgery)	Lab PSG	AHI ≥15	17 (41.5)	24 (58.5)		17 (41.5)		10
	40 (CABG)	Lab PSG	AHI ≥15	21 (52.5)	19 (47.5)		21 (52.5)		
Deflandre 2017 [43] (Belgium)	150 (Pre-operative patients; surgery type not specified))	Lab PSG	AHI > 5	134 (89.3)	16 (10.7)	41 (27.3)	30 (20)	63 (42)	9
Devaraj 2017 [44] (India)	182 (non-cardiac surgery)	HSAT (Resmed ApneaLink Plus)	AHI ≥ 5	70 (38.5)	112 (61.5)	59 (32.4)	11 (6)		13
Glazer 2018 [45] (Canada)	264 (bariatric surgery)	Lab PSG	AHI > 5	208 (78.8)	56 (21.2)	80 (30.3)	57 (21.6)	71 (26.9)	12
Horvath 2018 [46] (Switzerland)	251 (bariatric surgery)	HSAT (Embletta, Resmed)	AHI ≥ 5	109 (43.3)	142 (56.6)	57(22.7)	28 (11.2)	24 (9.6)	11
Spence 2018 [47] (United States)	Total: 84 With valid sleep study: 82 (THA/TKA)	HSAT (Natus Xltek or Watch-PAT200)	AHI ≥ 5	42 (51.2)	40 (48.8)	18 (21.2)	18 (21.2)	6 (7.3)	10
Kreitinger 2020 [48] (United States)	214 (bariatric surgery)	Lab PSG (*n* = 141) and HSAT (*n* = 73)	AHI ≥ 15	97 (45.3)	64 (29.9)	53 (24.8)	53 (24.8)	44 (20.6)	12
Lazaro 2020 [49] (Spain)	70 (bariatric surgery)	HSAT (ApneaLink vs10.20)	AHI ≥ 15	26 (37.1)	44 (62.8)		26 (37.1)		13
Waseem 2021- Caucasian [50] (Canada)	183 (non-cardiac surgery)	HSAT (ApneaLink Plus, Resmed)	AHI ≥ 15	63 (34.4)	46 (25.1)	74 (40.4)	38 (20.8)	25 (13.7)	13
Waseem 2021- Chinese [50] (Canada)	666 (non-cardiac surgery)	HSAT (ApneaLink Plus, Resmed	AHI ≥ 15	176 (26.4)	236 (35.4)	254 (38.1)	104 (15.6)	72 (10.8)	13
Waseem 2021- Indian [50] (Canada)	161 (non-cardiac surgery)	HSAT (ApneaLink Plus, Resmed	AHI ≥ 15	46 (28.6)	45 (28.0)	70 (43.5)	29 (18.0)	17 (10.6)	13
Waseem 2021- Malay [50] (Canada)	195 (non-cardiac surgery)	HSAT (ApneaLink Plus, Resmed	AHI ≥ 15	82 (42.1)	62 (31.8)	51 (26.2)	54 (27.7)	28 (14.4)	13

^a^Total QUADAS score range = 0–14. *Abbreviations: AHI* apnea-hypopnea index, *Bang* body mass index, age, neck circumference, gender, *BMI* body mass index, *CABG* coronary artery bypass graft surgery, *HSAT* home sleep apnea testing, *OSA* obstructive sleep apnea, *PSG* polysomnography, *QUADAS* quality assessment of diagnostic accuracy studies, *RDI* respiratory disturbance index, *STOP* snoring, tiredness, observed apnea and high blood pressure, *THA* total hip arthroplasty, *TKA* total knee arthroplasty

### Methodological quality of the included studies

The included studies had QUADAS scores ranging from 9 to 13, denoting a moderate risk of bias (Table 1). PSG or HSAT was used as a reference test in all included studies to determine the accuracy of the STOP-Bang questionnaire (Table 2). Three studies [42, 43, 45] exclusively used PSG, two [41, 48] used a mix of PSG and HSAT, and five [44, 46, 47, 49, 50] used HSAT. Although the standard for the diagnosis of OSA is PSG, there were no significant disparities between studies that used PSG or HSAT regarding prevalence of OSA (Table 2) and accuracy of the STOP-Bang questionnaire (Fig. 2).

The evaluation of internal and external validities of the included studies is summarized in Supplementary Tables S2 and S3. Regarding the internal validity, index and reference tests were blindly executed in five studies [41, 44, 45, 49, 50] and STOP-Bang scores were interpreted independently from clinical data in two studies [44, 50] (Supplementary Table S2). All but one study [43], which had unspecified inclusion criteria, fully described their inclusion and exclusion criteria. In all 10 studies, adequate information was provided to describe the study setting and the demographics of the surgical patients, including age, sex, and BMI. Although one study [43] did not randomly select patients for PSG, all studies applied the STOP-Bang questionnaire without pre-screening for OSA. Overall, there is a low risk of bias in subject selection for the reference test across the 10 studies.

### Accuracy of the STOP-Bang questionnaire in surgical patients

For the STOP-Bang questionnaire, the pooled predictive parameters of a score three or greater to screen for OSA in patients undergoing surgery are presented in Table 3 and Figs. 2 and 3. The prevalence of all, moderate-to-severe, and severe OSA was 65, 38, and 17% respectively. The pooled sensitivity of the STOP-Bang questionnaire was high at 85% (95%CI: 82, 88%, I^2^: 40.9%), 88% (95%CI: 85, 89%, I^2^: 85.7%), and 90% (95%CI: 87, 93%, I^2^: 86.9%) in screening for all, moderate-to-severe, and severe OSA, respectively. The pooled specificity was moderate at 47% (95%CI: 42, 52%, I^2^: 87.2%), 29% (95%CI: 27, 32%, I^2^: 89.9%), and 27% (95%CI: 25, 29%, I^2^: 93.1%) for all, moderate-to-severe, and severe OSA, respectively.

**Table 3:** Pooled predictive parameters of STOP-Bang ≥3 as cut-off for surgical patients

Predictive parameters	All OSA (AHI ≥5)	Moderate-to-Severe OSA (AHI ≥15)	Severe OSA (AHI ≥30)
	(4 studies, *n* = 1160)	(8 studies, *n* = 2812)	(5 studies, *n* = 2447)
Prevalence (%)	65 (62 to 68)	38 (36 to 40)	17 (16 to 19)
Sensitivity (%)	85 (82 to 88)	88 (85 to 89)	90 (87 to 93)
Specificity (%)	47 (42 to 52)	29 (27 to 32)	27 (25 to 29)
Positive predictive value (%)	74.9 (71.8 to 77.7)	42.9 (40.8 to 45.0)	20.3 (18.5 to 22.2)
Negative predictive value (%)	62.7 (56.9 to 68.1)	79.6 (76.2 to 82.6)	93.2 (90.9 to 95.1)
Diagnostic odds ratio	4.63 (2.76 to 7.75)	3.36 (2.67 to 4.21)	4.17 (2.74 to 6.35)
AUC	0.8437 SE = 0.0531	0.6729 SE = 0.0351	0.6302 SE = 0.0488
Likelihood ratio (+)	1.56 (1.21 to 2.01)	1.24 (1.16 to 1.32)	1.27 (1.20 to 1.34)
Likelihood ratio (−)	0.37 (0.30 to 0.46)	0.40 (0.31 to 0.52)	0.27 (0.14 to 0.52)
Post-test probability (%; positive test)	74.5%	42.9%	20.6%
Post-test probability (%; negative test)	40.9%	19.5%	5.2%

Data presented as means with 95% confidence interval in parentheses. *Abbreviations: AUC* area under the curve, *AHI* apnea–hypopnea index, *OSA* obstructive sleep apnea, *Bang* body mass index, age, neck circumference, gender, *SE* standard error, *STOP* snoring, tiredness, observed apnea and high blood pressure

The pooled positive predictive value (PPV) was highest at 75% (95%CI: 71.8, 77.7%) in detecting all OSA, and the corresponding PPVs for moderate-to-severe and severe OSA were 43% (95%CI: 40.8, 45.0%) and 20% (95%CI: 18.5, 22.2%), respectively (Table 3). The negative predictive value (NPV) for severe OSA was highest at 93.2% (95%CI: 90.9, 95.1%), indicating that the STOP-Bang questionnaire can reasonably rule-out severe OSA. A negative score of 0–2 would decrease the probability of diagnosing severe OSA from 17.0 to 5.2%. The corresponding NPV values for all and moderate-to-severe OSA were 62.7% (95% CI: 56.9, 68.1%) and 79.6% (95% CI: 76.2, 82.6). The area under the curve (AUC) was 0.84, 0.67, and 0.63 for all, moderate-to-severe, and severe OSA, respectively.

### Accuracy of different STOP-Bang score thresholds

The accuracy of different STOP-Bang score cut-offs in surgical patients for all OSA (*n* = 5722), moderate-to-severe OSA (*n* = 12,207) and severe OSA (*n* = 9878) are summarized in Supplementary Table S4. With the increase in the STOP-Bang threshold from 3 to 5, the sensitivity diminished from 88 to 50% for moderate-to-severe OSA and from 90 to 61% for severe OSA. As well, there was an increase in specificity from 29 to 78% for moderate-to-severe and from 27 to 75% for severe OSA. The PPV was highest at 86% with a STOP-Bang threshold of 6 or greater for detecting all OSA. Similarly, the NPV was highest for severe OSA at 94% for a threshold of 4 or greater.

### Meta-regression and sensitivity analysis

Meta-regression and sensitivity analysis were conducted for moderate-to-severe OSA with 12 study groups and for severe OSA in eight studies (Supplementary Tables S5 and S6). The analysis revealed that continuous variables marginally altered the combined estimates without significant effect on the results. Similarly, the categorical variables also marginally altered the combined estimates without significance. There was no significant effect on the results by any individual study as shown by leave-one-study-out analysis (Supplementary Fig. S1).

## Discussion

To date, our study is the first systematic review and meta-analysis examining the validity of the STOP-Bang questionnaire in the preoperative setting for screening of OSA in the surgical population. We demonstrate that a STOP-Bang score three or greater has excellent AUC of 0.84 to detect OSA in patients undergoing surgery. The high sensitivity and significant diagnostic odds ratio of STOP-Bang score ≥ 3 across the three OSA severities help identify patients undergoing surgery at increased risk for OSA. Similarly, the high NPV of 93.2% can help clinicians to reasonably exclude severe OSA in patients that score 0 to 2.

The prevalence of OSA in our study was high: 65, 38, and 17% for all, moderate-to-severe, and severe OSA, respectively. This is in keeping with previously reported prevalence of OSA in surgical patients [51, 52]. Overall, the high prevalence in the surgical versus the general population [53, 54] could be a consequence of higher burden of comorbidities in patients undergoing surgery, which may be risk factors for OSA.

There may be variations in the predictive accuracy of the STOP-Bang questionnaire within different ethnic groups. Devaraj et al. found a sensitivity of 82.8% and specificity of 65.2% [44]. A recent large prospective cohort study found that the optimal BMI cut-off in Indian population to be > 27.5 kg/m^2^ and STOP-Bang score 4 or greater as the optimal discrimination score to predict moderate-to-severe and severe OSA [50]. Most importantly, a recent meta-analysis on the performance of STOP-Bang in different geographic regions in 47 studies with 26,547 participants found it to be a valid screening tool worldwide [13]. Our study included patients across different countries and ethnicities, and our findings apply broadly to the surgical population.

In an ideal setting, every patient with undiagnosed OSA should be identified to minimize the risk of perioperative complications. Given limited logistical, financial, and clinical resources, especially in the preoperative setting, clinicians must carefully balance between the missed cases of OSA and the use of healthcare resource to diagnose OSA. In this regard, the predictive parameters of the screening tool are important measures for clinicians to take into consideration when screening patients. Sensitivity and specificity are two parameters that are typically inversely related. We found that an increase in the STOP-Bang cut-off corresponded to increased specificity and a reciprocal decrease in sensitivity in the detection of all, moderate-to-severe, and severe OSA. Surgical patients who score three or greater on STOP-Bang have a high probability of moderate-to-severe OSA [41, 55]. We found that a STOP-Bang threshold of six or greater had the highest PPV of 86% with high specificity of 90% for detecting all OSA (Supplementary Table S4). Our finding is consistent with a recent study that showed a STOP-Bang threshold of 6 has a high specificity of 91% in detecting OSA [56]. Whereas a STOP-Bang score of three or greater can be used to risk stratify patients at increased risk of OSA, a higher threshold may be useful for a patient population with a higher prevalence of OSA to reduce false-positives. In general, surgical patients should be screened with a threshold of three or greater unless a high prevalence of OSA is suspected, in which case a threshold of five or six may be beneficial to identify those at high-risk of undiagnosed OSA and in most need of further evaluation.

### Utility of the STOP-Bang questionnaire in patients undergoing surgery

Despite rising awareness and increase in prevalence of OSA in patients undergoing surgery, [53, 57] the vast majority of patients with OSA are unidentified preoperatively [2, 3, 52]. Undiagnosed OSA has been associated with difficult airway management [58] and increased postoperative complications including cardiovascular events, reintubation, respiratory complications, and longer hospital stay [2, 17, 59–61]. Notably, preoperative use of the STOP-Bang questionnaire to screen surgical patients to detect undiagnosed OSA has been shown to predict postoperative complications [17, 18, 28, 59, 62]. Of note, several of these studies are non-randomized, observational studies [60–62].

An increased severity of OSA may be associated with an increased rate of postoperative complications. Severe OSA was found to be associated with increased risk of postoperative cardiac complications [2]. Similarly, a higher incidence of postoperative complications was associated with higher OSA severity [63]. As higher STOP-Bang score is associated with higher risk of moderate-to-severe and severe OSA, [41] our findings indicate that the STOP-Bang questionnaire is a valid screening tool for preoperative risk stratification.

A recent study found that patients identified by the STOP-Bang questionnaire (score ≥ 3) as at increased risk for OSA had a 4-fold increase in post-operative cardiopulmonary events [59]. Similarly, patients with STOP-Bang score ≥ 3 experienced worse perioperative respiratory outcomes and prolonged hospital stay [62]. As such, missed awareness of OSA in surgical patients can put substantial strain on the healthcare system due to increased consumption of resources in the form of intensive care, increased ventilator support, and longer length of hospitalization [64]. Patients with OSA and compliant with their continuous positive airway pressure (CPAP) therapy were shown to have improved oxygen desaturation index on the night of surgery and were less likely to require oxygen therapy [65]. In addition, surgical patients with OSA and a CPAP prescription were associated with fewer cardiovascular complications, [17] further highlighting the importance of preoperative identification of undiagnosed OSA.

Nevertheless, limited time between preoperative evaluation and surgery, patient hesitance to undergo sleep testing, and long waitlists for sleep clinics are barriers to recognizing undiagnosed OSA. This underscores the importance of access to a robust and easy-to-administer screening tool with a high predictive accuracy. We report that that the STOP-Bang questionnaire is a valid screening tool that addresses this need with a high AUC of 0.84 for clinicians to risk-stratify preoperative patients and to plan mitigation for perioperative complications associated with OSA. Surgical patients at high risk of OSA should be considered for postoperative monitoring such as continuous oximetry and capnography [66, 67].

There are some limitations in our study. First, both PSG and HSAT were used as diagnostic tools for OSA in the included studies. Although the two are often equitable, some heterogeneity may be present as PSG is the diagnostic standard. Secondly, the internal validity was difficult to assess as blinding of the index and reference tests was unclear. Nevertheless, QUADAS tool was used to provide additional evaluation of the quality of the included studies. Lastly, our study population included a variety of surgical procedures, which may limit the applicability of our results to specific surgical populations. The combination of methodological variations in the diagnostic tools, the variability in prevalence of OSA across the studies, and the various surgical procedures likely resulted in high heterogeneity of the predictive parameters. In anticipation of this heterogeneity, a random-effects model was used for the meta-analysis. Nevertheless, our study presents a current review of the literature on the accuracy of the STOP-Bang questionnaire as a preoperative screening tool in the surgical population.

## Conclusions

In summary, our systematic review and meta-analysis demonstrates the validity of the STOP-Bang questionnaire for screening of OSA in surgical patients. With a score cut-off of 3 or greater, the STOP-Bang questionnaire has a high sensitivity and NPV, demonstrating its predictive utility to detect OSA in the surgical cohort.

## Supplementary Information

**Additional file 1: Supplementary Table S1.** Excluded studies and reasons for exclusion. **Supplementary Table S2.** Appraisal of the included studies based on criteria for internal validity. **Supplementary Table S3.** Appraisal of the included studies based on criteria for external validity. **Supplementary Table S4.** Predictive parameters of various STOP-Bang cut-offs for different OSA severities in surgical patients. **Supplementary Table S5.** Meta-regression and sensitivity analysis of various subgroups for AHI ≥ 15. **Supplementary Table S6.** Meta-regression and sensitivity analysis of various subgroups for AHI ≥ 30. **Supplementary Fig. S1.** Leave one study out analysis. **Appendix 1.** MEDLINE Search Strategy

## Data Availability

All data generated or analysed during this study are included in this published article and its supplementary information files.

## Abbreviations

AHI: apnea–hypopnea index
AUC: area under the curve
DOR: diagnostic odds ratio
HSAT: home sleep apnea testing
NPV: negative predictive value
OSA: obstructive sleep apnea
PPV: positive predictive value
PSG: polysomnography
PRISMA: preferred reporting items for systematic reviews and meta-analyses
PROSPERO: international prospective register of systematic reviews
QUADAS: quality assessment of diagnostic accuracy studies
RDI: respiratory disturbance index
