# Supplementary material for: Validation of the STOP-Bang questionnaire as a preoperative screening tool for obstructive sleep apnea: a systematic review and meta-analysis
Source: BMC Anesthesiol. 2022 Nov 30;22:366. doi: 10.1186/s12871-022-01912-1 (PMC9710034; doi:10.1186/s12871-022-01912-1)
Supplement: Supplementary file 1 — Additional file 1: Supplementary Table S1. Excluded studies and reasons for exclusion. Supplementary Table S2. Appraisal of the included studies based on criteria for internal validity. Supplementary Table S3. Appraisal of the included studies based on criteria for external validity. Supplementary Table S4. Predictive parameters of various STOP-Bang cut-offs for different OSA severities in surgical patients. Supplementary Table S5. Meta-regression and sensitivity analysis of various subgroups for AHI ≥ 15. Supplementary Table S6. Meta-regression and sensitivity analysis of various subgroups for AHI ≥ 30. Supplementary Fig. S1. Leave one study out analysis. Appendix 1. MEDLINE Search Strategy [file 12871_2022_1912_MOESM1_ESM.docx]

Table of Contents

[Supplementary Table S1. Excluded studies and reasons for exclusion 2](#_Toc94548690)

[Supplementary Table S2. Appraisal of the included studies based on criteria for internal validity 3](#_Toc94548691)

[Supplementary Table S3. Appraisal of the included studies based on criteria for external validity 4](#_Toc94548692)

[Supplementary Table S4. Predictive parameters of various STOP-Bang cut-offs for different OSA severities in surgical patients 5](#_Toc94548693)

[Supplementary Table S5. Meta-regression and sensitivity analysis of various subgroups for AHI ≥ 15 6](#_Toc94548694)

[Supplementary Table S6. Meta-regression and sensitivity analysis of various subgroups for AHI ≥ 30 7](#_Toc94548695)

[Supplementary Figure S1. Leave one study out analysis 8](#_Toc94548696)

[Appendix 1. MEDLINE Search Strategy 9](#_Toc94548697)

# Supplementary Table S1. Excluded studies and reasons for exclusion

| **Study** | **Exclusion Reason** |
| --- | --- |
| Vasu et al. 2010 | No sleep study |
| Lockhart et al. 2013 | Did not report predictive parameters |
| Kulkarni et al. 2014 | Did not report predictive parameters |
| Seet et al. 2015 | No sleep study |
| Dixon et al. 2016 | No sleep study |
| Reed et al. 2016 | Did not report predictive parameters |
| Bamgbade et al. 2017 | Did not report predictive parameters |
| Lakdawala et al. 2018 | No sleep study |
| Mason etn al. 2018 | No sleep study |
| Krishnasamy et al. 2019 | Did not report predictive parameters |
| Sankar et al. 2019 | No sleep study |
| Carr et al. 2020 | Did not report predictive parameters |
| Duarte et al. 2020 | Did not report predictive parameters |
| Kara et al. 2020 | No sleep study |
| Sangkum et al. 2021 | No sleep study |
| Spielmanns et al. 2021 | Did not report predictive parameters |

# Supplementary Table S2. Appraisal of the included studies based on criteria for internal validity

| Internal Criteria | Valid reference standard | Definition of the disease based on reference standard | Blind execution of index test and reference test | Index test interpreted independently of clinical information | Study design |
| --- | --- | --- | --- | --- | --- |
| Definition | **Laboratory PSG (Lab PSG) or**  **Home Sleep Apnea Testing (HSAT)** | **OSA diagnosed based on the PSG results** | **PSG readings blinded to the questionnaire results and vice versa** | **The questionnaire interpreted independently of clinical information** | **Prospective or Retrospective** |
| Chung et al. 2012 [37] | Standard PSG and HSAT | F | F | U | Prospective |
| Nunes et al. 2014 [38] | Lab PSG | F | N | U | Prospective |
| Deflandre et al. 2017 [39] | Lab PSG | F | N | U | Prospective |
| Devaraj et al. 2017 [40] | HSAT | F | F | F | Prospective |
| Glazer et al. 2018 [41] | Lab PSG | F | F | U | Retrospective |
| Horvath et al. 2018 [42] | Respiratory Polygraphy | F | N | U | Retrospective |
| Spence et al. 2018 [43] | HSAT | F | N | U | Prospective |
| Kreitinger et al. 2020 [44] | PSG and HSAT | F | N | U | Retrospective |
| Lazaro et al. 2020 [45] | HSAT | F | F | U | Prospective |
| Waseem et al. 2021 [46] | HSAT | F | F | F | Prospective |

F: Full meeting criteria; P: Partially meeting criteria; U: Unsure if meeting criteria in subgroups; not sure; N: Not meeting criteria in subgroups; N/A: Not applicable

# Supplementary Table S3. Appraisal of the included studies based on criteria for external validity

| External Criteria | Spectrum of diseases | Settings | Previous screening | Demographic information | Explication of cut-off point of index test | Percentage missing | Missing data management | Subject selection for reference test |
| --- | --- | --- | --- | --- | --- | --- | --- | --- |
| Definition | **Inclusion and exclusion criteria mentioned** | **Enough information to identify setting** | **No pre-screening before application of the questionnaire** | **Age, gender, BMI data provided** | **Results presented for AHI ≥5 or RDI≥15** | **Percentage missing mentioned** | **Analysis of missing data for basic characteristics** | **All subjects were invited or randomly selected to do PSG** |
| Chung et al. 2012 [37] | **F** | **F** | **F** | **F** | **F** | **F** | **F** | **F** |
| Nunes et al. 2014 [38] | **F** | **F** | **F** | **F** | **F** | **P** | **N** | **F** |
| Deflandre et al. 2017 [39] | **P** | **F** | **F** | **F** | **F** | **F** | **N** | **N** |
| Devaraj et al. 2017 [40] | **F** | **F** | **F** | **F** | **F** | **F** | **F** | **F** |
| Glazer et al. 2018 [41] | **F** | **F** | **F** | **F** | **F** | **N** | **N** | **F** |
| Horvath et al. 2018 [42] | **F** | **F** | **F** | **F** | **F** | **N** | **N** | **F** |
| Spence et al. 2018 [43] | **F** | **F** | **F** | **F** | **F** | **N** | **N** | **F** |
| Kreitinger et al. 2020 [44] | **F** | **F** | **F** | **F** | **F** | **N** | **N** | **F** |
| Lazaro et al. 2020 [45] | **F** | **F** | **F** | **F** | **F** | **F** | **N** | **F** |
| Waseem et al. 2021 [46] | **F** | **F** | **F** | **F** | **F** | **F** | **N** | **F** |

F: Full meeting criteria; P: Partially meeting criteria; U: Unsure if meeting criteria in subgroups; not sure; N: Not meeting criteria in subgroups; N/A: Not applicable

# Supplementary Table S4. Predictive parameters of various STOP-Bang cut-offs for different OSA severities in surgical patients

| **STOP-Bang Cut-offs** | **Number of study groups** | **Sample Size** | **Prevalence** | **Sensitivity** | **Specificity** | **PPV** | **NPV** |
| --- | --- | --- | --- | --- | --- | --- | --- |
| **All OSA** | | | | | | | |
| ≥ 3 | 4 | 1,160 | 65.2 (62.3 – 67.9) | 85 (82 – 88) | 47 (42 – 52) | 74.9 (71.8 – 77.7) | 62.7 (56.9 – 68.1) |
| ≥ 4 | 2 | 896 | 71.9(68.8- 74.8) | 65.5(61.7- 69.1) | 59.5(53.2- 65.6) | 80.5(76.8- 83.8) | 40.3(35.3- 45.5) |
| ≥ 5 | 3 | 978 | 70.2(67.1- 73) | 43.3(39.6- 47.1) | 79.4(74.2- 83.8) | 83.2(78.8- 86.8) | 37.3(33.5- 41.3) |
| ≥ 6 | 2 | 896 | 71.9(68.8- 74.8) | 23.4(20.2- 27) | 90.4(86- 93.7) | 86.3(80- 90.8) | 31.6(28.2- 35.1) |
| ≥ 7 | 2 | 896 | 71.9(68.8- 74.8) | 10(7.9- 12.7) | 92(87.8- 95) | 76.5(65.6- 84.7) | 28.6(25.5- 31.9) |
| ≥ 8 | 2 | 896 | 71.8(68.8- 74.8) | 1.7(0.9- 3.1) | 98.8(96.2- 99.9) | 78.6(48.8- 94.3) | 28.2(25.3- 31.3) |
| **Moderate-to-severe OSA** | | | | | | | |
| ≥ 3 | 12 | 2,812 | 37.7 (35.9 – 39.5) | 88 (85 – 89) | 29 (27 – 32) | 42.9 (40.8– 45) | 79.6 (76.2 – 82.6) |
| ≥ 4 | 10 | 2,648 | 35.3(33.5- 37.1) | 74.1(71.1- 76.8) | 55.7(53.3- 58) | 47.7(45.1- 50.1) | 79.8(77.4- 82) |
| ≥ 5 | 10 | 2,478 | 36.6(34.7- 38.5) | 49.7(46.4- 53) | 78(75.8- 80) | 56.5(53- 60) | 72.9(70.7- 75) |
| ≥ 6 | 9 | 2,396 | 36.8(34.9- 38.8) | 25.1(22.4- 28.2) | 92.4(90.9- 93.7) | 65.9(60.5- 70.9) | 67.9(65.9- 69.9) |
| ≥ 7 | 4 | 977 | 42.8(39.7- 50) | 12.9(9.9- 16.6) | 96.2(94.2- 97.6) | 72(60.3- 81.5) | 59.6(56.4- 62.8) |
| ≥ 8 | 2 | 896 | 42.4(39.1- 45.7) | 4.2(2.5- 6.8) | 98.6(97- 99.4) | 69.6(47- 85.9) | 58.3(54.9- 61.6) |
| **Severe OSA** | | | | | | | |
| ≥ 3 | 8 | 2,447 | 17 (15.5 – 18.6) | 90 (87 – 93) | 27 (25 – 29) | 20.3 (18.5 – 22.2) | 93.2 (90.9 – 95.1) |
| ≥ 4 | 6 | 2,101 | 16(14.6- 17.8) | 83.1(78.7- 86.9) | 50.8(48.4- 3.1) | 24.5(22.1- 27.1) | 94(92.2- 95.3) |
| ≥ 5 | 7 | 2,183 | 15.8(14.3- 17.4) | 61.4(56- 66.5) | 75.1(73- 77) | 31.6(28.2- 35.4) | 91.2(89.6- 92.5) |
| ≥ 6 | 6 | 2,101 | 16.1(14.6- 17.8) | 33.3(28.4- 38.7) | 90.7(89.2- 92) | 40.9(35.1- 47) | 87.6(86- 89) |
| ≥ 7 | 2 | 896 | 22(19.3- 24.9) | 19.8(14.6- 26.1) | 95.4(93.5- 96.8) | 54.9(42.7- 66.6) | 80.8(78- 83.4) |
| ≥ 8 | 1 | 150 | 42(34- 50) | 7.9(2.9- 18.2) | 97.7(91.1- 99.6) | 71.4(30.2- 94.9) | 59.4(50.9- 67.5) |

# Supplementary Table S5. Meta-regression and sensitivity analysis of various subgroups for AHI ≥ 15

|  | **Sensitivity** | | | **Specificity** | | | **Diagnostic Odds Ratio- Log scale** | | |
| --- | --- | --- | --- | --- | --- | --- | --- | --- | --- |
| **Co-variate**  **(Number of study groups)** | **Point Estimate**  **(95% CI)** | **Coefficient (SE)** | **p-value** | **Point Estimate**  **(95% CI)** | **Coefficient (SE)** | **p-value** | **Point Estimate**  **(95% CI)** | **Coefficient (SE)** | **p-value** |
| **Apnea Hypopnea Index (AHI) ≥ 15 (12)** | | | | | | | | | |
| Age (12) | 90.1 (83.7- 94.1) | -0.00 (0.05) | 0.87 | 28.8 (22- 36.7) | 0.00 (0.02) | 0.71 | 1.21 (0.98- 1.43) | -0.00 (0.05) | 0.91 |
| Male gender (12) | 90.1 (83.7- 94.1) | 0.28 (0.16) | 0.07 | 28.8 (22- 36.7) | -0.07 (0.10) | 0.46 | 1.21 (0.98- 1.43) | 0.20 (0.19) | 0.29 |
| BMI (12) | 90.1 (83.7- 94.1) | 1.62 (1.17) | 0.16 | 28.8 (22- 36.7) | 0.05 (0.72) | 0.95 | 1.21 (0.98- 1.43) | 1.58 (1.41) | 0.26 |
| Neck circumference (12) | 90.1 (83.7- 94.1) | -4.77 (3.89) | 0.22 | 28.8 (22- 36.7) | -0.31 (2.36) | 0.90 | 1.21 (0.98- 1.43) | -4.70 (4.66) | 0.31 |
| Sample Size (12) | 90.1 (83.7- 94.1) | 0.00 (0.00) | 0.34 | 28.8 (22- 36.7) | 0.00 (0.00) | 0.48 | 1.21 (0.98- 1.43) | 0.00 (0.00) | 0.27 |
| Prevalence (12) | 90.1 (83.7- 94.1) | 0.16 (0.14) | 0.24 | 28.8 (22- 36.7) | 0.04 (0.09) | 0.67 | 1.21 (0.98- 1.43) | 0.18 (0.17) | 0.29 |
| Study type  • Prospective (10)  • Retrospective (02) | 90 (87.7- 91.9)  76 (70- 82) | 0.18 (1.70) | 0.91 | 27.5 (22.9- 32.7)  43 (37- 49) | -0.59 (0.67) | 0.38 | 1.23 (0.97- 1.49)  3.44 (2.64-  4.47) * | -1.33 (1.87) | 0.47 |
| Validation tool  • Lab PSG (04)  • Home PSG (06)  • Both (02) | 78 (73- 83)  89.7 (86.2- 92.3)  91 (87- 93) | 3.97 (5.32)  8.12 (7.45) | 0.45  0.27 | 43 (36- 49)  29.4 (23.7- 35.9)  29 (25- 33) | 2.55 (3.24)  2.58 (4.63) | 0.43  0.59 | 2.86 (1.84-  4.45) *  1.18 (0.82- 1.54)  3.91 (2.65-  5.77) * | 5.89 (6.41)  9.77 (9.06) | 0.35  0.28 |

*Pooled Diagnostic Odds Ratio (Model results)

Abbreviations: CI, confidence interval; SE, standard error.

# Supplementary Table S6. Meta-regression and sensitivity analysis of various subgroups for AHI ≥ 30

|  | **Sensitivity** | | | **Specificity** | | | **Diagnostic Odds Ratio- Log scale** | | |
| --- | --- | --- | --- | --- | --- | --- | --- | --- | --- |
| **Co-variate**  **(Number of study groups)** | **Point Estimate**  **(95% CI)** | **Coefficient (SE)** | **p-value** | **Point Estimate**  **(95% CI)** | **Coefficient (SE)** | **p-**  **value** | **Point Estimate**  **(95% CI)** | **Coefficient (SE)** | **p-**  **value** |
| **Apnea Hypopnea Index (AHI) ≥ 30 (8)** | | | | | | | | | |
| Age (8) | 93.3 (82.4- 97.7) | 0.09 (0.18) | 0.59 | 28 (20.7- 36.7) | -0.04 (0.03) | 0.12 | 1.39 (1.00- 1.78) | 0.04 (0.18) | 0.89 |
| Male gender (8) | 93.3 (82.4- 97.7) | 0.16 (0.24) | 0.62 | 28 (20.7- 36.7) | -0.02 (0.05) | 0.73 | 1.39 (1.00- 1.78) | 0.14 (0.23) | 0.57 |
| BMI (8) | 93.3 (82.4- 97.7) | 0.64 (0.93) | 0.49 | 28 (20.7- 36.7) | 0.01 (0.18) | 0.94 | 1.39 (1.00- 1.78) | 0.66 (0.94) | 0.49 |
| Neck circumference (8) | 93.3 (82.4- 97.7) | -1.94 (2.99) | 0.52 | 28 (20.7- 36.7) | -0.15 (0.56) | 0.79 | 1.39 (1.00- 1.78) | -2.11 (3.04) | 0.49 |
| Sample Size (8) | 93.3 (82.4- 97.7) | -0.00 (0.00) | 0.59 | 28 (20.7- 36.7) | -0.000 (<0.001) | 0.45 | 1.39 (1.00- 1.78) | -0.00 (0.00) | 0.45 |
| Prevalence (8) | 93.3 (82.4- 97.7) | 0.03 (0.02) | 0.16 | 28 (20.7- 36.7) | -0.00 (0.00) | 0.55 | 1.39 (1.00- 1.78) | 0.03 (0.02) | 0.21 |
| Study type  • Prospective (07)  • Retrospective (01) | 95 (92.1- 96.9)  64.8 (52.5- 75.5) | -2.81 (0.76) | <0.001 | 24.7 (20.8- 29)  56.5 (49.2- 63.5) | 1.91 (0.38) | <0.001 | 1.80 (1.29- 2.31) | -0.90 (0.83) | 0.28 |
| Validation tool  • Lab PSG (2)  • Home PSG (05)  • Both (1) | 80 (72- 86)  94.6 (89.4- 97.4)  94.8 (89.1- 97.7) | 0.52 (0.82)  -0.03 (0.54) | 0.53  0.96 | 43.9 (38.1- 50)  25.2 (20.4- 30.8)  27.6 (24.1- 31.4) | -0.69 (0.36)  -0.16 (0.19) | 0.06  0.40 | 2.81 (1.42- 5.57) *  1.68 (0.93- 2.43) | -0.17 (0.87)  -0.26 (0.55) | 0.85  0.64 |

*Pooled Diagnostic Odds Ratio (Model results)

Abbreviations: CI, confidence interval; SE, standard error.

# Supplementary Figure S1. Leave one study out analysis

**Moderate-to-severe OSA (AHI>=15)**


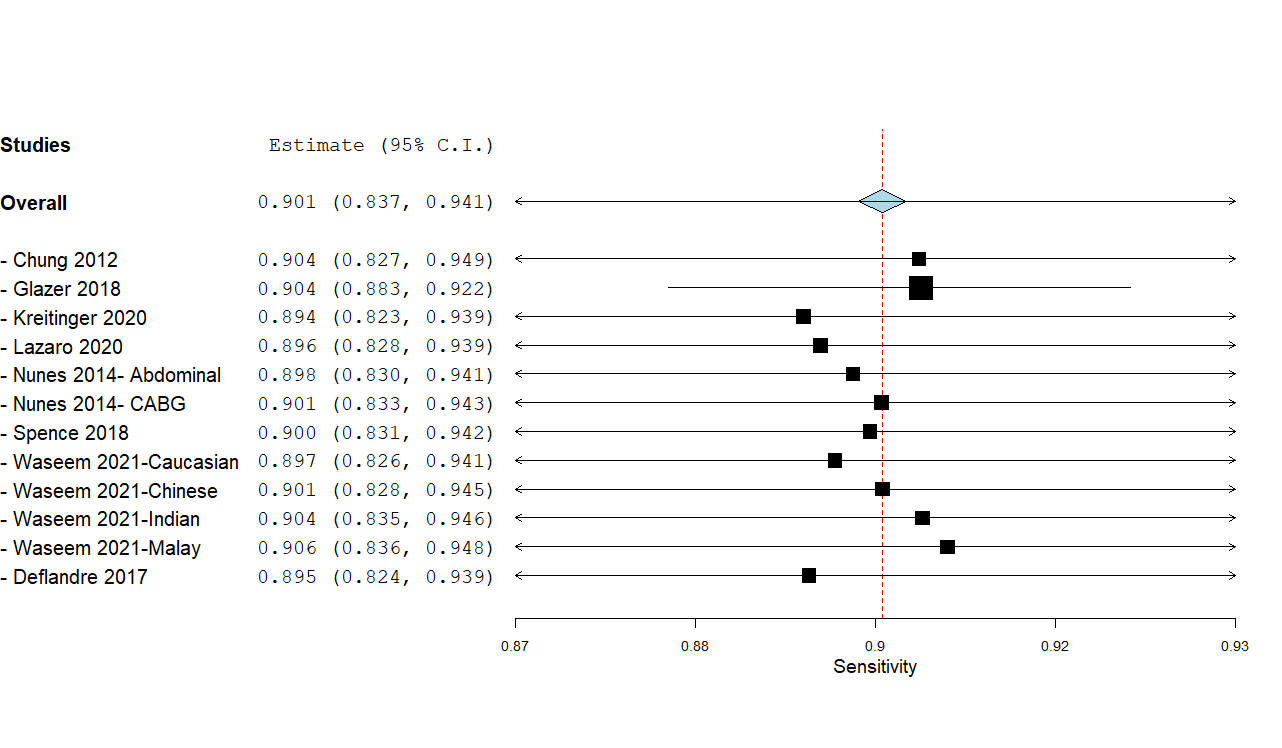


**Severe OSA (AHI>=30)**


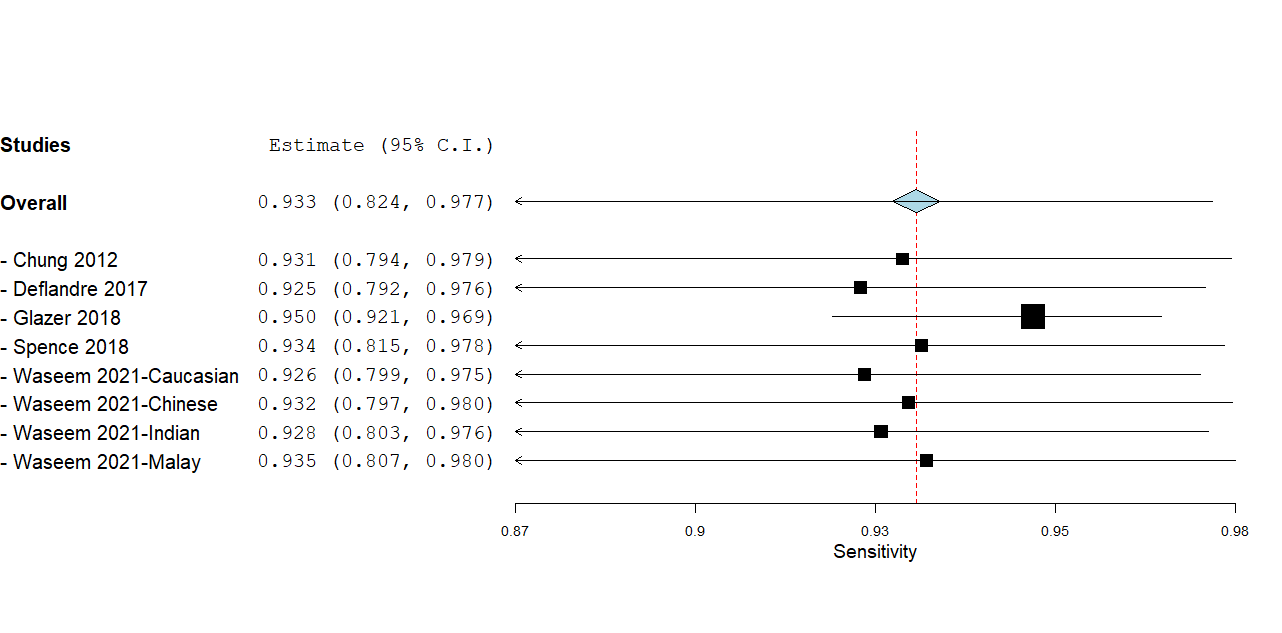


# Appendix 1. MEDLINE Search Strategy

Date completed: May 13, 2021

| **#** | **Searches** | **Results** |
| --- | --- | --- |
| 1 | "stop-bang".mp,kw,kf. | 2154 |
| 2 | "stopbang".mp,kw,kf. | 258 |
| 3 | "stop-bang".af. | 2268 |
| 4 | "stopbang".af. | 260 |
| 5 | 1 or 2 or 3 or 4 | 2411 |
| 6 | remove duplicates from 5 | 1583 |
| 7 | "Delayed Emergence from Anesthesia"/ | 545 |
| 8 | Anesthesia Recovery Period/ | 19331 |
| 9 | co.xs. and post*.mp. [Complications exploded floating subheading] | 299921 |
| 10 | exp "Anesthesia and Analgesia"/ | 1384189 |
| 11 | exp Intraoperative Complications/ | 118589 |
| 12 | exp Intraoperative Period/ | 299904 |
| 13 | exp Postoperative Care/ | 193335 |
| 14 | exp Postoperative Complications/ | 1518748 |
| 15 | exp Postoperative Period/ | 778649 |
| 16 | exp Specialties, Surgical/ | 6919318 |
| 17 | exp Surgeons/ | 234219 |
| 18 | exp Surgical Procedures, Operative/ | 10076420 |
| 19 | Operating Room Nursing/ | 16985 |
| 20 | Operating Rooms/ | 69198 |
| 21 | Operative Blood Salvage/ | 1538 |
| 22 | Operative Time/ | 135977 |
| 23 | Perioperative Care/ | 87336 |
| 24 | Perioperative Medicine/ | 370 |
| 25 | Perioperative Nursing/ | 14780 |
| 26 | Perioperative Period/ | 78134 |
| 27 | Postanesthesia Nursing/ | 2127 |
| 28 | Postoperative Cognitive Complications/ | 2205 |
| 29 | Reoperation/ | 202718 |
| 30 | Salvage Therapy/ | 45810 |
| 31 | su.fs. [ Surgery floating subheading ] | 4381539 |
| 32 | tr.fs. [ Transplant floating subheading ] | 148935 |
| 33 | (after adj6 (operation? or operative??)).mp,kw. | 388246 |
| 34 | (after adj6 (surgery or surgeries or surgical*)).mp,kw. | 1238387 |
| 35 | (aneurysm* adj2 repair*).mp,kw. | 40329 |
| 36 | (cardiac adj2 catheter* adj2 ablat*).mp,kw. | 410 |
| 37 | (carotid adj2 endarterectom*3).mp,kw. | 42412 |
| 38 | (consequen* adj6 (surgery or surgeries or surgical*)).mp,kw. | 16176 |
| 39 | (coronary adj2 catheter* adj2 ablat*).mp,kw. | 44 |
| 40 | (coronary adj2 radiofrequenc* adj2 ablat*).mp,kw. | 27 |
| 41 | (coronary adj3 bypass*).mp,kw. | 221001 |
| 42 | (coronary adj3 catheteri*).mp,kw. | 6892 |
| 43 | (follow* adj6 (operation? or operative??)).mp,kw. | 99848 |
| 44 | (follow* adj6 (surgery or surgeries or surgical*)).mp,kw. | 424736 |
| 45 | (heart adj2 radiofrequenc* adj2 ablat*).mp,kw. | 85 |
| 46 | (heart adj2 valve? adj2 repair*).mp,kw. | 344 |
| 47 | (heart adj2 valve? adj2 replac*).mp,kw. | 20607 |
| 48 | (hybrid-maze adj2 procedure*).mp,kw. | 13 |
| 49 | (mitral valve? adj2 repair*).mp,kw. | 23017 |
| 50 | (mitral valve? adj2 reparat*).mp,kw. | 32 |
| 51 | (near adj2 miss*).mp,kw. | 8736 |
| 52 | (open adj1 reduction?).mp,kw. | 44292 |
| 53 | (perioperat* or peri-operat*).mp,kw. | 381771 |
| 54 | (post* adj3 pain).mp,kw. | 266700 |
| 55 | (readmi* or re-admi*).mp,kw. | 186003 |
| 56 | (respiratory adj2 aspirat*).mp,kw. | 3184 |
| 57 | (subsequent* adj6 (operation? or operative??)).mp,kw. | 12360 |
| 58 | (subsequent* adj6 (surgery or surgeries or surgical*)).mp,kw. | 48248 |
| 59 | (transmyocardia* adj3 revasculari*).mp,kw. | 1693 |
| 60 | (transmyocardia* adj5 ablat*).mp,kw. | 11 |
| 61 | adenectom*.mp,kw. | 964 |
| 62 | adrenalectom*.mp,kw. | 69352 |
| 63 | an?esthe*.mp,jw,in. | 2165904 |
| 64 | angioplast*3.mp,kw. | 215354 |
| 65 | appendectom*.mp,kw. | 49046 |
| 66 | arthrectom*.mp,kw. | 130 |
| 67 | arthroplast???.mp,kw. | 278852 |
| 68 | atherectom*3.mp,kw. | 13306 |
| 69 | CABG.mp,kw. | 67929 |
| 70 | caesarean*.mp,kw. | 77678 |
| 71 | cardiac ablat*.mp,kw. | 1112 |
| 72 | cardiomyoplast*3.mp,kw. | 2764 |
| 73 | cervicectom*.mp,kw. | 930 |
| 74 | cesarean*.mp,kw. | 239878 |
| 75 | cholecystectom*.mp,kw. | 121546 |
| 76 | colectom*.mp,kw. | 52435 |
| 77 | coronary artery bypass surger*.mp,kw. | 40751 |
| 78 | cystectom*.mp,kw. | 60337 |
| 79 | cytokine release syndrome?.mp,kw. | 7805 |
| 80 | delayed emergence.mp,kw. | 1414 |
| 81 | discectom*.mp,kw. | 23454 |
| 82 | diskectom*.mp,kw. | 18529 |
| 83 | duodenectom*.mp,kw. | 2303 |
| 84 | emergence deliri???.mp,kw. | 1858 |
| 85 | esophagectom*.mp,kw. | 34756 |
| 86 | fundectom*.mp,kw. | 293 |
| 87 | gastrectom*.mp,kw. | 139319 |
| 88 | gerionco*.mp,kw. | 1 |
| 89 | glossectom*.mp,kw. | 4876 |
| 90 | gonadectom*.mp,kw. | 11668 |
| 91 | hemiarthroplast???.mp,kw. | 11774 |
| 92 | hemi-arthroplast???.mp,kw. | 579 |
| 93 | hemicolectom*.mp,kw. | 19219 |
| 94 | hemi-colectom*.mp,kw. | 477 |
| 95 | hepatectom*.mp,kw. | 82587 |
| 96 | hypophysectom*.mp,kw. | 36567 |
| 97 | iridectom*.mp,kw. | 5602 |
| 98 | jejunectom*.mp,kw. | 254 |
| 99 | laparoscop*.mp,kw. | 485272 |
| 100 | laparotom*.mp,kw. | 205962 |
| 101 | laryngectom*.mp,kw. | 32148 |
| 102 | lobectom*.mp,kw. | 82070 |
| 103 | lumpectom*.mp,kw. | 14310 |
| 104 | lymphadenectom*.mp,kw. | 69707 |
| 105 | lymph-adenectom*.mp,kw. | 215 |
| 106 | lymphectom*.mp,kw. | 129 |
| 107 | mandibulectom*.mp,kw. | 2626 |
| 108 | mastectom*.mp,kw. | 136418 |
| 109 | mastoidectom*.mp,kw. | 11515 |
| 110 | maxillectom*.mp,kw. | 4320 |
| 111 | mesohepatectom*.mp,kw. | 193 |
| 112 | meso-hepatectom*.mp,kw. | 6 |
| 113 | metastasectom*.mp,kw. | 7903 |
| 114 | MIDCAB.mp,kw. | 1249 |
| 115 | myectom*.mp,kw. | 5055 |
| 116 | myomectom*.mp,kw. | 16665 |
| 117 | necrosectom*.mp,kw. | 3833 |
| 118 | nephrectom*.mp,kw. | 144306 |
| 119 | neurosurg*.mp,kw. | 229700 |
| 120 | obstetr*.mp,in,kw,kf. | 1378104 |
| 121 | obstetr*.mp,kw. | 477163 |
| 122 | oesophagectom*.mp,kw. | 5282 |
| 123 | oncosurg*.mp,kw. | 669 |
| 124 | onco-surg*.mp,kw. | 167 |
| 125 | oncosurg*.mp. | 669 |
| 126 | onco-surg*.mp. | 167 |
| 127 | oophorectom*.mp,kw. | 30988 |
| 128 | operati*.mp,kw. | 3151994 |
| 129 | orchidectom*.mp,kw. | 8527 |
| 130 | orchiectom*.mp,kw. | 44925 |
| 131 | pancreatectom*.mp,kw. | 42536 |
| 132 | pancreaticoduodenectom*.mp,kw. | 40754 |
| 133 | pancreatico-duodenectom*.mp,kw. | 797 |
| 134 | parathyroidectom*.mp,kw. | 25812 |
| 135 | para-thyroidectom*.mp,kw. | 41 |
| 136 | peroperativ*.mp,kw. | 100504 |
| 137 | pharyng*esophagectom*.mp,kw. | 363 |
| 138 | pharyngectom*.mp,kw. | 2546 |
| 139 | pharyngolaryngoesophagectom*.mp,kw. | 299 |
| 140 | pneumonectom*.mp,kw. | 46856 |
| 141 | post*an?esth*.mp,kw. | 15975 |
| 142 | post-*an?esth*.mp,kw. | 9765 |
| 143 | post*cardiac.mp,kw. | 2468 |
| 144 | post*ectom*.mp,kw. | 39092 |
| 145 | post-*ectom*.mp,kw. | 510 |
| 146 | post*laparoscop*.mp,kw. | 403 |
| 147 | post-*laparoscop*.mp,kw. | 1267 |
| 148 | post*laparotom*.mp,kw. | 189 |
| 149 | post-*laparotom*.mp,kw. | 322 |
| 150 | post*microsurg*.mp,kw. | 6 |
| 151 | post-*microsurg*.mp,kw. | 21 |
| 152 | post-*operat*.mp,kw. | 298170 |
| 153 | post*ostom*.mp,kw. | 738 |
| 154 | post-*otom*.mp,kw. | 114 |
| 155 | post*pericardiot*.mp,kw. | 1254 |
| 156 | post*pex*.mp,kw. | 53 |
| 157 | post-*pex*.mp,kw. | 23 |
| 158 | post*plast*.mp,kw. | 3315 |
| 159 | post-*plast*.mp,kw. | 72 |
| 160 | post-*procedur*.mp,kw. | 37396 |
| 161 | post*reconstruct*.mp,kw. | 723 |
| 162 | post-*reconstruct*.mp,kw. | 1098 |
| 163 | post*scop*.mp,kw. | 2439 |
| 164 | post-*scop*.mp,kw. | 168 |
| 165 | post-*surg*.mp,kw. | 71624 |
| 166 | post*surger*.mp,kw. | 18144 |
| 167 | post-*surger*.mp,kw. | 40454 |
| 168 | post*transplant*.mp,kw. | 52407 |
| 169 | post-*transplant*.mp,kw. | 95545 |
| 170 | postintervention*.mp,kw. | 37474 |
| 171 | post-intervention*.mp,kw. | 83737 |
| 172 | postoperat*.mp,kw. | 2514567 |
| 173 | post-operat*.mp,kw. | 298165 |
| 174 | postpericardio*.mp,kw. | 1271 |
| 175 | post-pericardio*.mp,kw. | 391 |
| 176 | postproced*.mp,kw. | 24607 |
| 177 | postprocedur*.mp,kw. | 24596 |
| 178 | post-procedur*.mp,kw. | 37392 |
| 179 | postsurg*.mp,kw. | 61721 |
| 180 | post-surg*.mp,kw. | 71547 |
| 181 | proctocolectom*.mp,kw. | 14268 |
| 182 | prostatect*.mp,kw. | 126192 |
| 183 | quadrantectom*.mp,kw. | 1737 |
| 184 | re-*operat*.mp,kw. | 20476 |
| 185 | reoperat*.mp,kw. | 253405 |
| 186 | re-operat*.mp,kw. | 20475 |
| 187 | reresect*.mp,kw. | 551 |
| 188 | resect*.mp,kw. | 1218717 |
| 189 | rhinectom*.mp,kw. | 305 |
| 190 | salpingectom*.mp,kw. | 10758 |
| 191 | salpingo-oophorectom*.mp,kw. | 15414 |
| 192 | second look*.mp,kw. | 16429 |
| 193 | second-look*.mp. | 16429 |
| 194 | segmentectom*.mp,kw. | 14156 |
| 195 | sequelae.mp,kw. | 196733 |
| 196 | splenectom*.mp,kw. | 90570 |
| 197 | subsegmentectom*.mp,kw. | 663 |
| 198 | sub-segmentectom*.mp,kw. | 48 |
| 199 | surgeon*.mp,kw. | 726546 |
| 200 | surger*.mp,kw. | 7661852 |
| 201 | surger???.mp,kw. | 7661117 |
| 202 | surgical*.mp,kw. | 3994250 |
| 203 | takeback?.mp. | 118 |
| 204 | take-back?.mp. | 1211 |
| 205 | thymectom*.mp,kw. | 26994 |
| 206 | thyroidectom*.mp,kw. | 89186 |
| 207 | tonsillectom*.mp,kw. | 36900 |
| 208 | transfusion*.mp,kw. | 528540 |
| 209 | transplant*.mp,kw. | 1992432 |
| 210 | trauma*.mp,kw. | 1377045 |
| 211 | trisegmentectom*.mp,kw. | 991 |
| 212 | tri-segmentectom*.mp,kw. | 35 |
| 213 | tumorectom*.mp,kw. | 2846 |
| 214 | unanticipat*.mp,kw. | 23440 |
| 215 | undesired.mp,kw. | 24524 |
| 216 | unintended.mp,kw. | 50147 |
| 217 | uretectom*.mp,kw. | 25 |
| 218 | uvulectom*.mp,kw. | 263 |
| 219 | vaginectom*.mp,kw. | 936 |
| 220 | vulvectom*.mp,kw. | 4137 |
| 221 | exp surgery/ [ embase ] | 6827125 |
| 222 | exp surgeon/ | 223683 |
| 223 | or/7-222 [ PERIOPERATIVE OR SURGERY OR POSTOPERATIVE INCLUDING SURGERY OR POST*Ectom* ] | 19023856 |
| 224 | 5 and 223 | 1193 |
| 225 | remove duplicates from 224 | 768 |
| 226 | 225 use medall | 256 |
| 227 | 225 use emczd | 431 |
| 228 | 225 use psyh | 34 |
| 229 | 225 use emcr | 14 |
| 230 | 225 use cctr | 33 |
| 231 | 225 use coch | 0 |
